# Lysosome Evanescence Mediates Autophagic Flux Impairment in Glucose Imbalanced Environments

**DOI:** 10.1002/kjm2.70145

**Published:** 2025-12-13

**Authors:** Yuan‐Chen Cheng, Ling‐Li Chang, Hung‐Chen Wang, Wei‐Chun Hung, Chia‐Hua Hsieh, Yi‐Hsuan Wang, Kuang‐I Cheng

**Affiliations:** ^1^ Postgraduate Year Residency, Department of Medical Education, Chang Gung Memorial Hospital‐Kaohsiung Medical Center Chang Gung University College of Medicine Kaohsiung Taiwan; ^2^ School of Medicine, College of Medicine Kaohsiung Medical University Kaohsiung Taiwan; ^3^ Department of Microbiology and Immunology, College of Medicine Kaohsiung Medical University Kaohsiung Taiwan; ^4^ Department of Neurosurgery, Chang Gung Memorial Hospital‐Kaohsiung Medical Center Chang Gung University College of Medicine Kaohsiung Taiwan; ^5^ Department of Anesthesiology Kaohsiung Medical University Hospital, Kaohsiung Medical University Kaohsiung Taiwan

**Keywords:** autophagy, glucose variability, Lamp‐2, LC3 puncta, Schwann cell

## Abstract

Schwann cells (SCs) support axonal function and promote nerve regeneration. This study investigated how various glucose concentrations influence SC viability, oxidative stress, and autophagy, which contribute to diabetic neuropathy. RSC96 SCs were cultured under five glucose conditions (0, 2.5, 5.5, 50, or 100 mM) for 24, 48, and 72 h. Cell viability was assessed by MTT; ROS levels were determined by DCFDA staining, and apoptosis and ER stress markers, as well as autophagy‐related proteins were assessed by Western blotting. Autophagic flux was also analyzed using bafilomycin A1 and Cyto‐ID. The results revealed time‐dependent increases in cell death across all glucose conditions, especially under deprivation and low glucose (LG) conditions. ROS, CHOP, Bax, and cleaved caspase‐3 levels increased from 24 to 72 h. The initial increase in the LC3B‐II/I ratio indicated that autophagy was overactivated under glucose deprivation or LG conditions but impaired by 72 h, as indicated by reduced Lamp‐2 expression. High glucose conditions led to early and persistent autophagy suppression, with minimal autophagic flux and vacuole formation. These findings demonstrate that SCs are sensitive to glucose levels and undergo distinct autophagic impairments through different mechanisms under persistent low or high glucose environments.

**Abbreviations**
AMPK: AMP‐activated protein kinase
BSA: bovine serum albumin
DAPI: 4',6‐diamidino‐2‐phenylindole
DPN: diabetic peripheral neuropathy
DM: diabetic mellitus
ER: endoplasmic reticulum
h: hour
HG: high glucose
Lamp‐2: lysosomal‐associated membrane protein 2
LG: low glucose
PNS: peripheral nervous system
ROS: reactive oxygen species
SCs: Schwann cells
SE: standard error
SEM: standard error mean
WB: Western blot

## 1 Introduction

The pathogenesis of diabetic peripheral neuropathy (DPN) is complex. Persistent hyperglycemia, considered the initiating factor of diabetes, promotes the accumulation of advanced glycation end products, increases in reactive oxygen species (ROS) generation, and induces both microvascular and macrovascular complications [1]. Tight glycemic control remains difficult for diabetes mellitus (DM) patients who use conventional insulin therapy, as hypoglycemia and glucose fluctuations contribute to complications [2]. Rapid decreases in blood glucose, especially below 2.1 mM, can critically reduce cerebral glucose levels leading to significant brain injury [3]. Severe or recurrent hypoglycemia is associated with neurodegeneration, retinopathy, and cardiovascular events [4]. In the peripheral nerve system (PNS), prolonged mild hypoglycemia can cause Wallerian‐like axonal degeneration starting at nerve terminals, with somatic motor nerves more strongly affected by hypoglycemia, while hyperglycemia mainly impacts somatic sensory nerves [5].

Schwann cells (SCs), the main glial cells in the PNS, are essential for neuronal structure, function, and repair, and their dysfunction is a key factor in DPN pathogenesis [6]. SCs exposed to high glucose (HG) show reduced viability, elevated ROS levels, and NLRP3 inflammasome activation, all of which contribute to DPN progression [7]. In mammalian models, hyperglycemia‐induced oxidative damage leads to axonal degeneration, demyelination, and apoptosis, resulting in the loss of both myelinated and unmyelinated fibers [8]. Hyperglycemia may also impair SC differentiation and myelination via ERK signaling activation [9]. However, compared with sustained hyperglycaemia, glucose variability may pose a greater risk for diabetes‐related complications than sustained hyperglycemia, as recurrent glucose fluctuations induce oxidative stress and apoptosis in SCs, with in vitro studies showing significantly reduced cell viability under alternating glucose levels [10]. Clinically, recurrent hypoglycemia is linked to increased hospital readmission and mortality.

Autophagy, which is critical for energy balance and cell survival, plays a key role in DPN and has been linked to axonal degeneration and regeneration during hypoglycemia [11, 12], although its role in insulin‐induced hypoglycemia remains unclear. Given that the nervous system relies almost exclusively on glucose oxidation for ATP production, and that SCs are highly sensitive to shifts in glucose levels, understanding the effects of glucose deprivation is essential. While hyperglycemia‐induced SC dysfunction has been well studied, little is known about nutrient‐deficient conditions. This study aims to clarify the metabolic vulnerability of SCs and their role in DPN progression by investigating how varying glucose concentrations affect SC viability, oxidative stress, ER stress, apoptosis, and autophagy.

## 2 Materials & Methods

### 2.1 Cell Culture and Reagents

The rat Schwann cell line RSC96 (BCRC#60507, Taiwan) was obtained from the Bioresource Collection and Research Center and cultured in DMEM supplemented with 10% fetal bovine serum, 100 mg/mL streptomycin, and 100 U/mL penicillin at 37°C in a humidified incubator with 5% CO_2_. To investigate the concentration‐dependent effects of glucose, cells were exposed to normal glucose (5.5), glucose deprivation (0), low glucose (2.5), or HG (50 and 100 mM) for 24, 48, or 72 h (h) to assess induction of cytotoxicity, oxidative stress, and autophagy induction. Bafilomycin A1 (1nM; Sigma‐Aldrich, B1793) was used to inhibit lysosomal acidification and block autophagic flux, allowing the measurement of autophagic degradation activity, while rapamycin (500 nM; Sigma‐Aldrich, 553210) was used to induce autophagy.

### 2.2 Cell Viability Assay

The viability of RSC96 was evaluated using MTT (Abcam, ab146345) assays. For the assays, cells were seeded at 2 × 10^4^ cells/well in 12‐well plates and cultured under glucose deprivation, low, or high glucose conditions for 24, 48, and 72 h, respectively. The mitochondrial dehydrogenase activity of living cells was measured via MTT. A minimum of three independent assays, using separate cell batches, was performed for each experimental group.

### 2.3 Detection of Intracellular ROS

RSC96 cells were cultured in 96‐well plates for total ROS assessment. Induced levels of intracellular ROS were evaluated using a DCFDA/H2DCFDA‐Cellular ROS Assay Kit (Abcam, ab113851) according to the manufacturer's instructions. Three independent assays, using separate cell batches, were performed for each experimental group.

### 2.4 Western Blots

Cultured SCs were lysed in RIPA buffer (50 mM Tris, pH 7.4, 150 mM NaCl, 1 mM EDTA, 0.1% sodium dodecyl sulfate, 1% NP‐40, and 0.5% sodium deoxycholate) supplemented with cOmplete protease inhibitor cocktail tablets (Roche, 11697498001). Protein concentrations were measured using the Bradford reagent assay kit (Bio‐Rad, B6916) supplemented with bovine serum albumin (BSA). Equal amounts (20 μg) of protein were separated on 8%–12% SDS‐PAGE gels and transferred to polyvinylidene fluoride membranes (Merck Millipore, IPVH00010). The membranes were probed with primary antibodies against CHOP (Cell Signaling Technology, L63F7), Bax (Abcam, ab32503), caspase‐III (Novus Biologicals, NB100‐56708), LC3 (Cell Signaling Technology, 3868), P62 (Novus Biologicals, 42841), Lamp‐2 (Invitrogen, PA1‐655), and β‐actin (Millipore Sigma, MAB1501) followed by horseradish peroxidase‐conjugated goat anti‐mouse (Merck, AP124P) or goat anti‐rabbit (Merck, AP132P) secondary antibodies. Protein bands were visualized using Amersham ECL Select WB detection reagents (Cytiva, RPN2235) and imaged with a UVP ChemiDoc‐ItR 810 system (P/N 97–0645–05, Cambridge CB4 1TG, UK; UVP97062301). The target protein levels were normalized to those of tβ‐actin and quantified relative to the normal glucose control. Each group was assessed in at least three independent experiments using separate cell batches. A full list of antibodies and dilutions is provided in Table 1.

**TABLE 1:** Key resource table.

Reagent or resource	Source	Identifiers
Antibodies (dilution)		
Bax (1:1000)	Abcam	Ab32503
ß‐Actin (1:5000)	Millipore Sigma	MAB1501
Caspase‐III (1:1000)	Novus Biologicals	42,841
CHOP (1:500)	Cell Signaling Technology	L63F7
LAMP‐2 (1:1000)	Invitrogen	PA1‐655
LC3 (1:500)	Cell Signaling Technology	3868
P62 (1:500)	Novus Biologicals	42,841
HRP‐conjugated goat anti‐mouse secondary antibody (1:1000)	Merck	AP124P
HRP‐conjugated goat anti‐rabbit secondary antibody (1:1000)	Merck	AP132P
AlexaFluor488‐conjugated goat anti‐rabbit secondary antibody (1:1000)	Thermo Fisher Scientific	A11008
Chemicals and assay kits		
Amersham ECL Select Western blot detection reagents	Cytiva	RPN2235
Bafilomycin A_1_	Sigma‐Aldrich	B1793
Bradford Reagent Protein Assay Kit	Bio‐Rad	B6916
cOmplete protease inhibitor cocktail tablets	Roche	11,697,498,001
Cyto‐ID Autophagy Detection kit	Enzo Life Sciences	ENZ175
DCFDA/H2DCFDA‐Cellular ROS Assay Kit	Abcam	ab113851
MTT (3‐[4,5‐dimethylthiazol‐2‐yl]‐2,5 diphenyl tetrazolium bromide)	Abcam	ab146345
Rapamycin	Sigma‐Aldrich	553,210
VECTASHIELD HardSet Antifade Mounting Medium with DAPI	Vector Laboratories	H‐1500‐10
Instrument and software		
ChemiDoc‐ItR 810 imaging system	UVP	UVP97062301
FluoView️ FV1000 Confocal Imaging system	Olympus	
Keynote v.14.4 (7043.0.93)	Apple2003–2025	
Leica DMi8 fluorescence microscope	Leica	
SPSS20	IBM	

### 2.5 Immunofluorescence

Immunofluorescence analysis was performed to detect LC3 puncta and Lamp‐2 lysosome expression in SCs under various glucose concentrations. Briefly, cells were seeded onto collagen‐coated coverslips in 6‐well plates and fixed with 4% paraformaldehyde for 20 min at room temperature. Following fixation, the cells were washed with phosphate‐buffered saline (PBS), then blocked and permeabilized for 1 h in 3% BSA in PBS containing 0.1% Triton X‐100. The cells were then incubated overnight at 4°C with either a rabbit anti‐LC3 primary antibody (Cell Signaling Technology; 3868) or a rabbit anti‐Lamp‐2 primary antibody (Invitrogen, PA1‐655). For secondary antibody staining, the cells were incubated for up to 3 h at room temperature with AlexaFluor 488‐conjugated goat anti‐rabbit secondary antibody (Thermo Fisher Scientific, #A11008). Finally, the samples were washed, then mounted in Vectashield (Vector laboratories, H‐1500‐10) and imaged using a Leica DMi8 fluorescence microscopy system. A full list of the antibodies and assay dilution details can be found in Table 1.

### 2.6 CYTOID Autophagy Fluorescence Staining

CYTO‐ID Autophagy Detection Kit (Enzo Life, ENZ175) was used to monitor autophagic vesicle accumulation, which appears as bright fluorescence within cells. The cells were seeded at a density of 1 × 10^5^ cells/ml in a 12‐well plate and cultured for 24, 48, and 72 h under various glucose conditions. Briefly, after cell adhesion, the cells were incubated with CYTO‐ID staining solution (Enzo Life Sciences, ENZ175) in the dark at 37°C for 30 min. Following staining, Hoechst 33,342 blue fluorescent dye was applied for nuclear labeling prior to mounting in Vectashield (H‐1500‐10). Cells were observed using the Olympus FV 1000 Confocal Microscope system to assess autophagic vesicle formation. Acquired images were assessed and processed using the FluoView FV1000 Imaging system and Imaris Viewer 9.9.0 (Oxford instruments).

### 2.7 Statistical Analysis

The results are presented as the standard error of the mean (SEM), with any error bar representing ± standard error (SE) from at least three independent experiments. Group comparisons were performed using one‐way ANOVA, with a *p* value < 0.05 considered to indicate statistical significance using IBM SPSS Statistics 20.

## 3 Results

### 3.1 Glucose‐Limiting or Glucose‐Deprivation Conditions Exacerbated Cell Death

**FIGURE 1:**
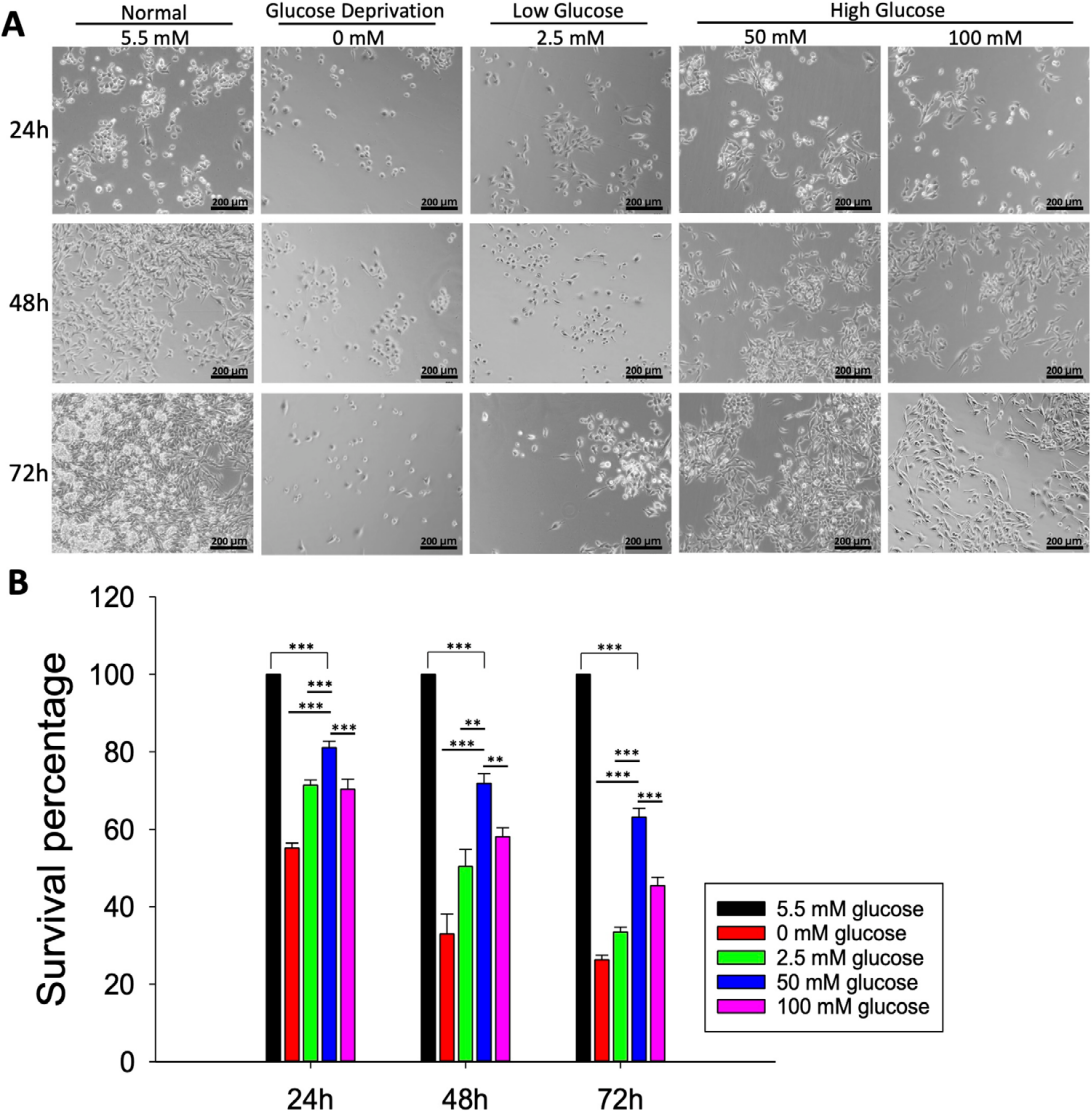
Viabilities of Schwann cells under different glucose concentrations were determined by MTT. (A) Representative images of RSC96 cultured in 0, 2.5, 5.5, 50, or 100 mM glucose‐containing medium for 24, 48, and 72 h. (B) Levels of cell viability were detected via MTT assays (5× independent assays per group). Bars represent means ±SEs. Statistical analyses were performed via one‐way ANOVA, with *p < 0.05, **p < 0.01, and ***p < 0.001. Scale bar: 200 μm.

RSC96 cells were cultured and incubated with varying glucose concentrations, and the cell viability of each treatment group was assessed via an MTT assay. After being cultured under normal conditions, cells in the 5.5 mM glucose control group exhibited robust proliferation and remained in a healthy state after 72 h of adherent culture; these cells served as the normal control (Figure 1A).

Compared with that of the normal glucose control, cell viability progressively decreased over 24–72 h under glucose deprivation, LG, and 2 HG conditions in a time‐dependent manner (Figure 1B). After 24 h, compared with those under the 5.5 mM glucose condition, cell viability decreased to 55% under glucose deprivation, 71% under LG, and 70%–81% under HG conditions compared to 5.5 mM glucose. After 72 h, the cell viability further decreased to 26% and 33% under glucose deprivation and LG conditions, respectively. In contrast, compared with normal control cells, cells under HG conditions maintained 45%–63% viability (Figure 1B).

Within the HG groups, cell viability decreased progressively as the glucose concentration increased. Furthermore, compared with 50 mM HG conditions, glucose deprivation and LG conditions resulted in significantly lower viability (Figure 1B). Overall, cell viability decreased in a time‐dependent manner, with glucose deprivation and LG conditions having a more pronounced effect than HG exposure did.

### 3.2 Altered Glucose Conditions Upregulate Oxidative Stress and ER Stress

**FIGURE 2:**
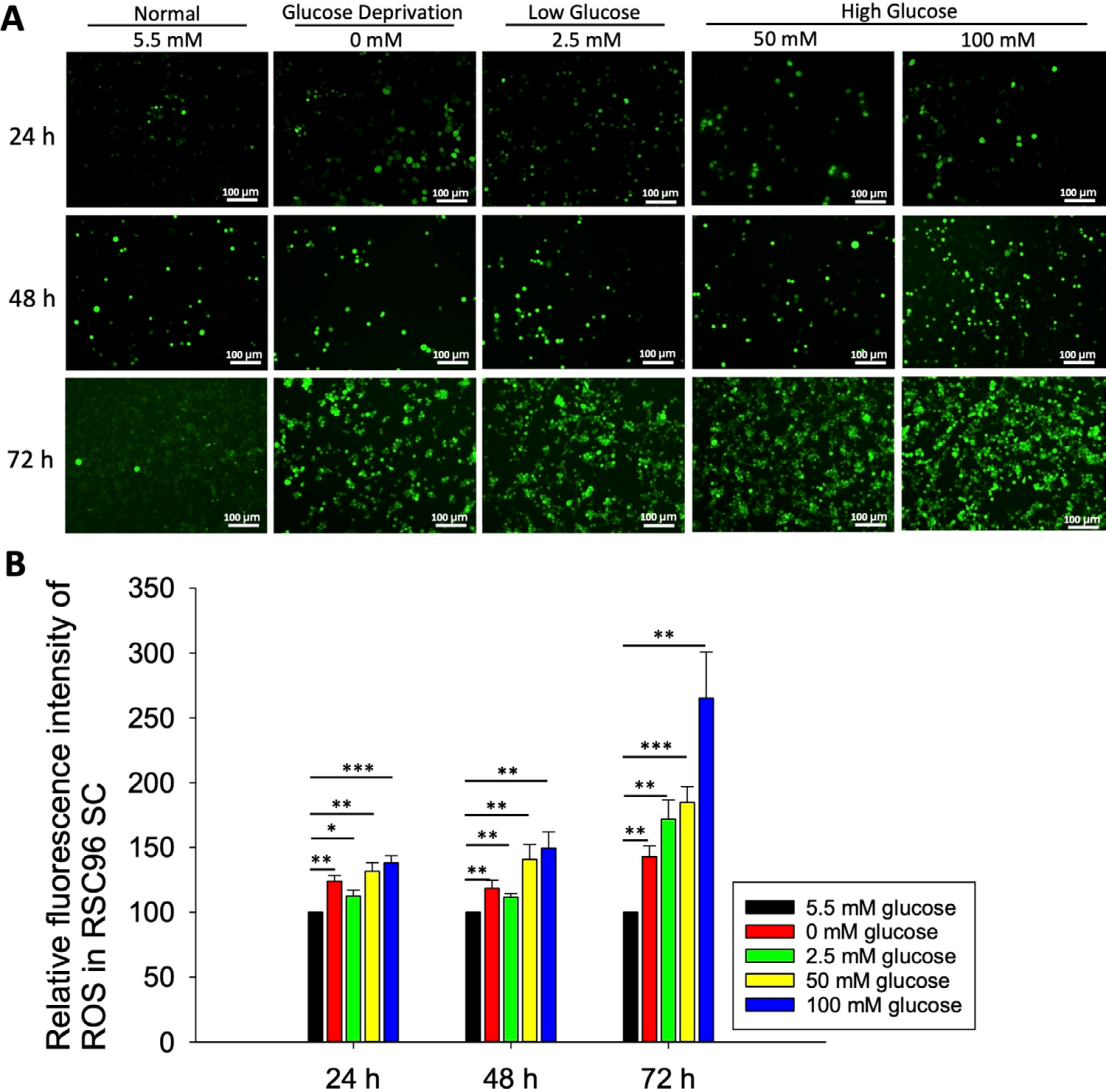
ROS induced in Schwann cells under different glucose concentrations. (A) Representative images of ROS within RSC96 cultured in 0, 2.5, 5.5, 50, or 100 mM glucose‐containing medium for 24, 48, and 72 h. Intracellular ROS generation was determined by DCFDA staining. (B) Fluorescence intensities correspond to levels of ROS in the differentially conditioned cells (4× independent assays per group). Bars represent means ±SEs. Statistical analyses between the 5.5 mM glucose control group and each respective glucose treatment group per time point were performed via one‐way ANOVA, with *p < 0.05, **p < 0.01, ***p < 0.001. Scale bar: 100 μm.

**FIGURE 3:**
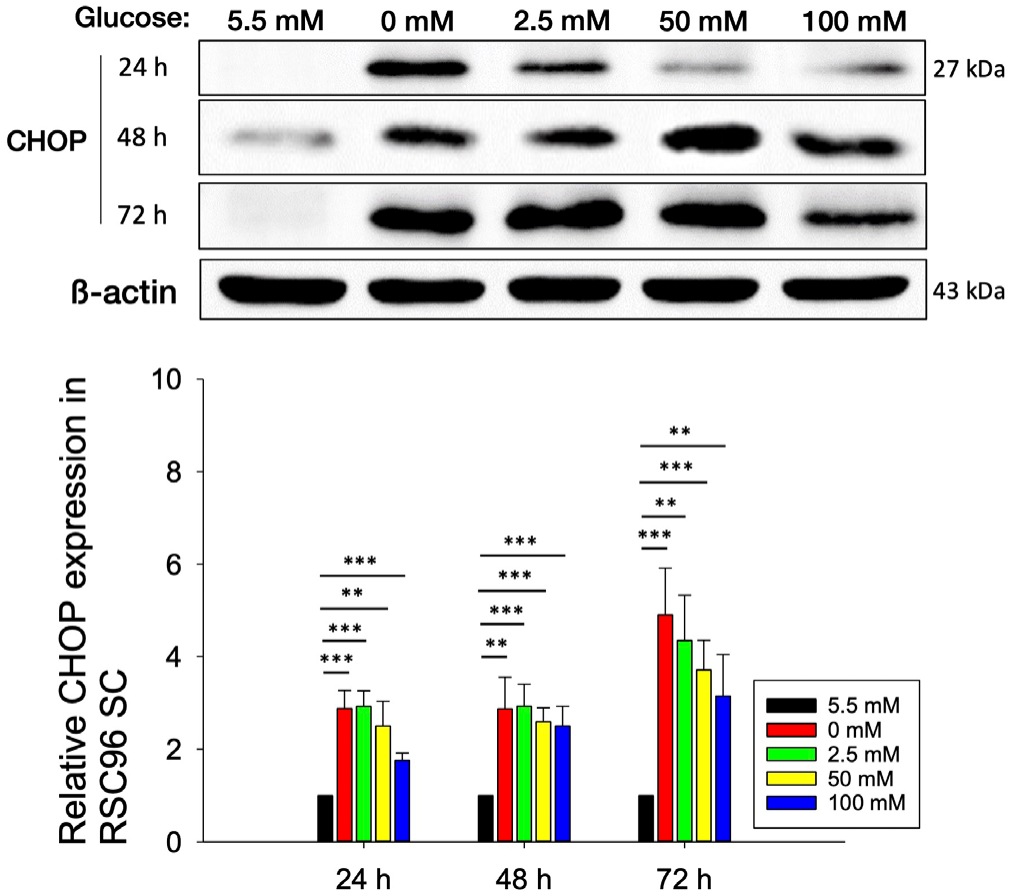
Endoplasmic reticulum stress was induced in Schwann cells under different glucose concentrations. RSC96 cells were cultured in 0, 2.5, 5.5, 50, or 100 mM glucose‐containing medium for 24, 48, and 72 h. The expression levels of CHOP were detected and evaluated via WB (4× independent assays per group). Bars represent means ±SEs. Statistical analyses between the 5.5 mM glucose control group and each respective glucose treatment group per time point were performed via one‐way ANOVA, with *p < 0.05, **p < 0.01, ***p < 0.001.

ROS levels in RSC96 cells cultured under normal glucose, glucose deprived, LG or HG conditions were measured using the DCFDA/H_2_DCFDA Cellular ROS Assay Kit, where the fluorescence intensities corresponded to the intracellular ROS levels (Figure 2A). Compared with those in the 5.5 mM glucose control group, the ROS levels were slightly but significantly elevated at 24 h, and further increased by 72 h across all the glucose treatment groups (Figure 2A,B). In lieu of apparent ROS elevation, endoplasmic reticulum (ER) stress was evaluated on the basis of CHOP protein expression levels, a transcription factor associated with the ER stress response, particularly under glucose imbalanced conditions, as validated by Western blot (WB) analysis [13, 14]. Results showed that exposure to glucose deprivation, LG, or HG significantly increased CHOP expression in a time‐dependent manner, with increases detectable as early as 24 h and sustained through 72 h (Figure 3). Although the ER stress response appeared to be stronger in the glucose‐deprived and LG groups than in the HG group, the differences were not statistically significant.

### 3.3 Prolonged Glucose Imbalances Induced Apoptosis

**FIGURE 4:**
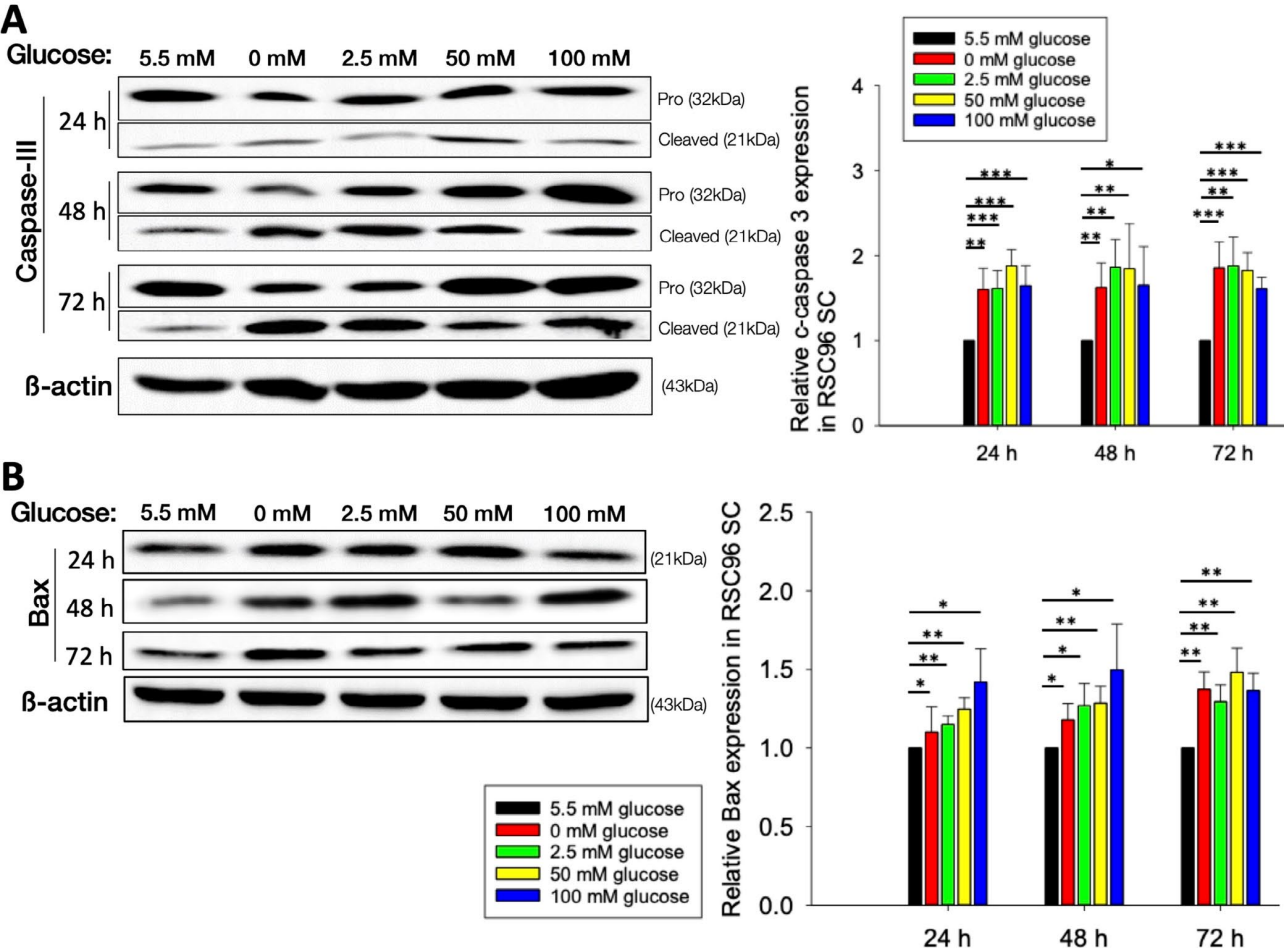
Elevated levels of cellular apoptosis in Schwann cells under different glucose concentrations. Expression levels of the pro‐apoptosis markers (A) cleaved‐caspase‐3 and (B) Bax in RSC96 cultured in 0, 2.5, 5.5, 50, or 100 mM glucose‐containing medium for 24, 48, and 72 h (4× independent assays per group). Bars represent means ±SEs. Statistical analysis between the 5.5 mM glucose control group and each respective glucose treatment group per time point was performed via one‐way ANOVA, with *p < 0.05, **p < 0.01, ***p < 0.001.

Caspase‐3 is a key mediator of oxidative stress‐induced apoptosis, whereas Bax, a pro‐apoptotic member of the Bcl‐2 protein family, promotes mitochondrial membrane permeabilization and activates the intrinsic apoptotic pathway. WB analysis revealed that the levels of cleaved caspase‐3 (Figure 4A) and Bax (Figure 4B) were significantly greater under glucose deprivation, LG, and HG conditions than under the control at 24, 48, and 72 h, indicating that abnormal glucose levels consistently trigger apoptotic signaling over time.

### 3.4 Glucose Imbalance Impaired Autophagy via Distinct Mechanisms

**FIGURE 5:**
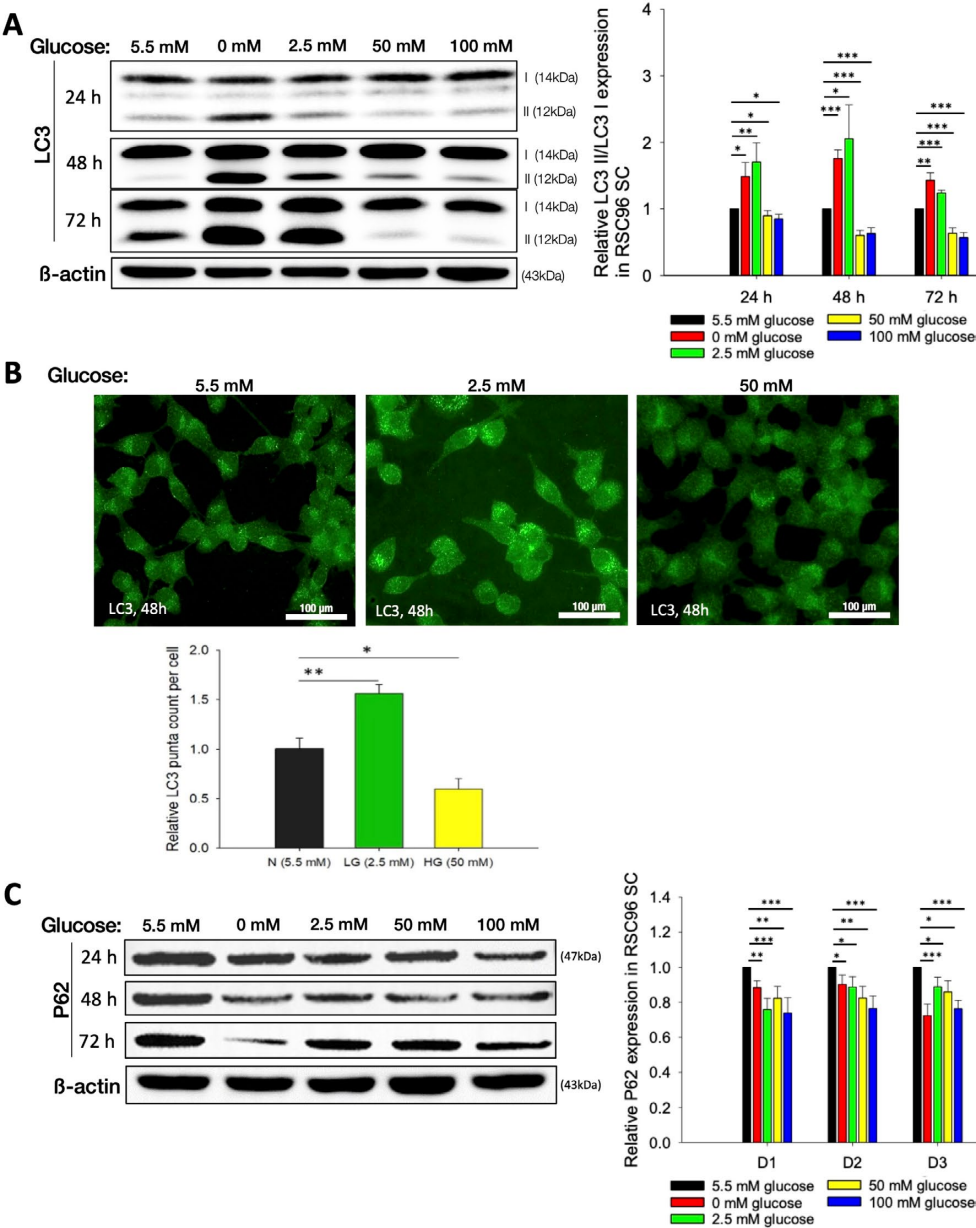
Differentially levels of LC3 protein expressions in Schwann cells under different glucose concentrations. WB analysis of (A) LC3‐I and ‐II expressions in RSC96 cultured in 0, 2.5, 5.5, 50, or 100 mM glucose‐containing medium for 24, 48, and 72 h. WB data expresses in ratio of LC3‐II/LC3‐I levels normalized with the 5.5 mM glucose group as normal control. (B) Representative images of fluorescent intracellular LC3 puncta in RSC96 (green). (C) WB analysis of p62 protein expressions. Bars represent means ± SEs. Statistical analysis between the 5.5 mM glucose control group and each respective glucose treatment group per time point were performed via one‐way ANOVA, with *p < 0.05, **p < 0.01, ***p < 0.001. Scale bar: 100 μm (4× independent assays per group).

WB analysis revealed that the expression ratio of LC3B‐II/‐I, a marker of autophagosome accumulation, was significantly increased in RSC96 SCs cultured under glucose deprivation and LG conditions at 24, 48, and 72 h, indicating enhanced autophagosome formation. In contrast, HG conditions led to significantly lower LC3B‐II/‐I ratios at all time points, suggesting the suppression of autophagosome formation (Figure 5A). Fluorescence imaging further supported these findings, as distinct LC3‐positive puncta were detected in both LG‐ and HG‐treated cells at 48 h (Figure 5B). Compared with normal glucose, P62, an autophagy adaptor protein degraded during autophagic flux, was reduced across all glucose‐altered conditions at 24, 48, and 72 h compared to normal glucose, suggesting active autophagic degradation despite variations in autophagosome formation (Figure 5C).

**FIGURE 6:**
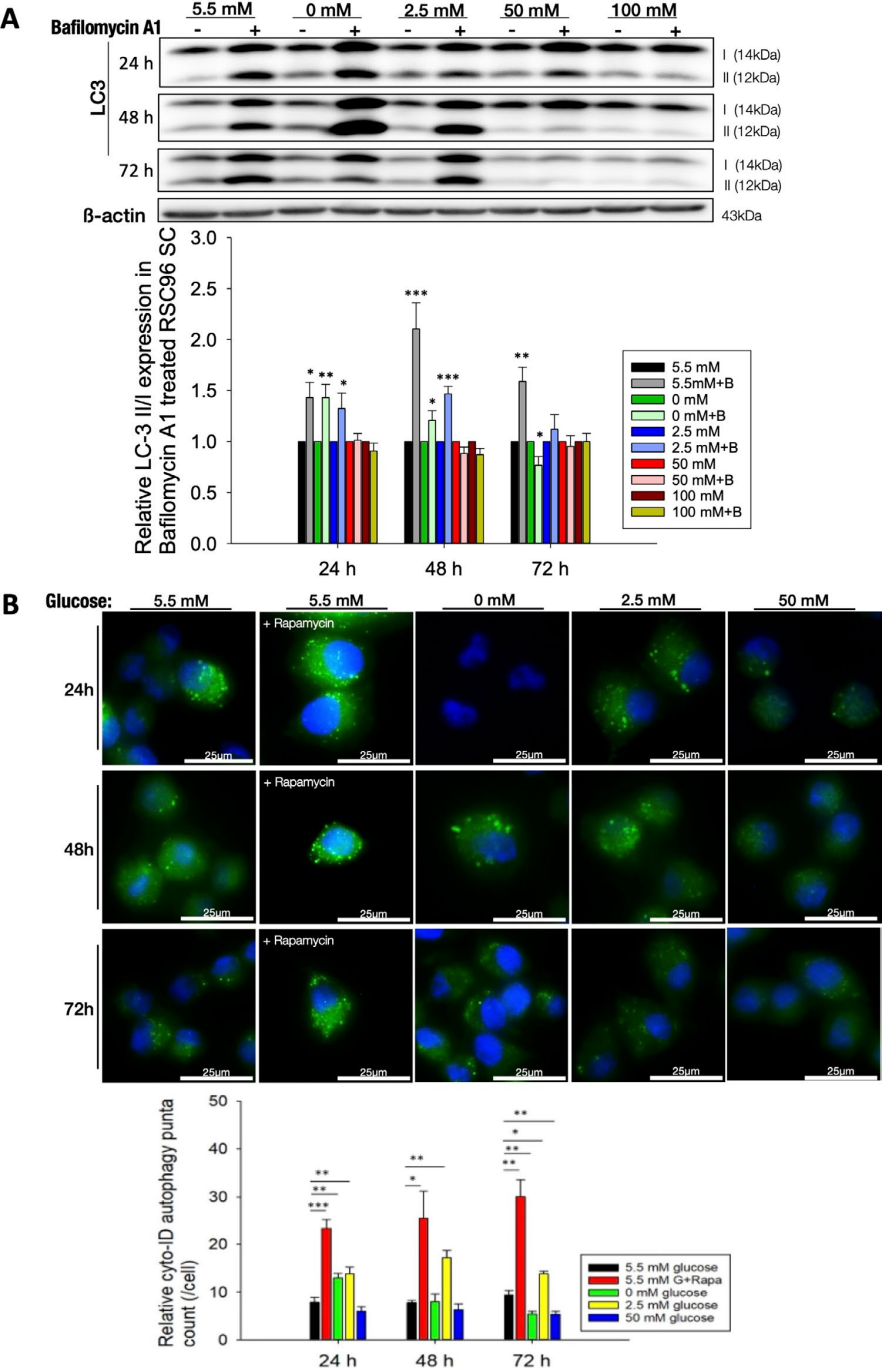
Autophagy flux evaluation in Schwann cells under different glucose concentrations. (A) WB analysis of LC3‐II/LC3‐I expressions in cells under various glucose conditions treated with or without the lysosomal inhibitor, bafilomycin‐A1 (B; 1 nM). Bars represent means ± SEs of 4–6 independent experiments. (B) Representative images of CYTO ID autophagy fluorescence assays demonstrating intracellular autophagic compartments (green puncta) and DAPI (4′,6‐diamidino‐2‐phenylindole)‐stained nuclei. Statistical analysis between each glucose treatment group and it's respective bafilomycin‐A1 treatment group were performed via one‐way ANOVA, with *p < 0.05, **p < 0.01, ***p < 0.001. Scale bar: 25 μm.

Under normal glucose conditions, bafilomycin A1 treatment led to significant increases in the LC3B‐II/‐I ratio at all time points, confirming active autophagic flux (Figure 6A). Under glucose‐deprived and LG conditions, flux was only elevated at 24 and 48 h, but not at 72 h, indicating a decrease in autophagic activity over time. No significant changes were observed under HG conditions regardless of treatment, suggesting sustained suppression of autophagic flux. To further examine the effects of LG and HG imbalances on autophagic flux abilities in SCs, the CYTO‐ID Autophagy Detection Kit was used to measure autophagic vacuoles that showed vivid fluorescence in cells [15]. The results of the assays revealed increased autophagic vesicle accumulation in glucose‐deprived and LG‐treated SCs at 24 and 48 h, and consistently reduced staining in HG‐treated cells (Figure 6B).

**FIGURE 7:**
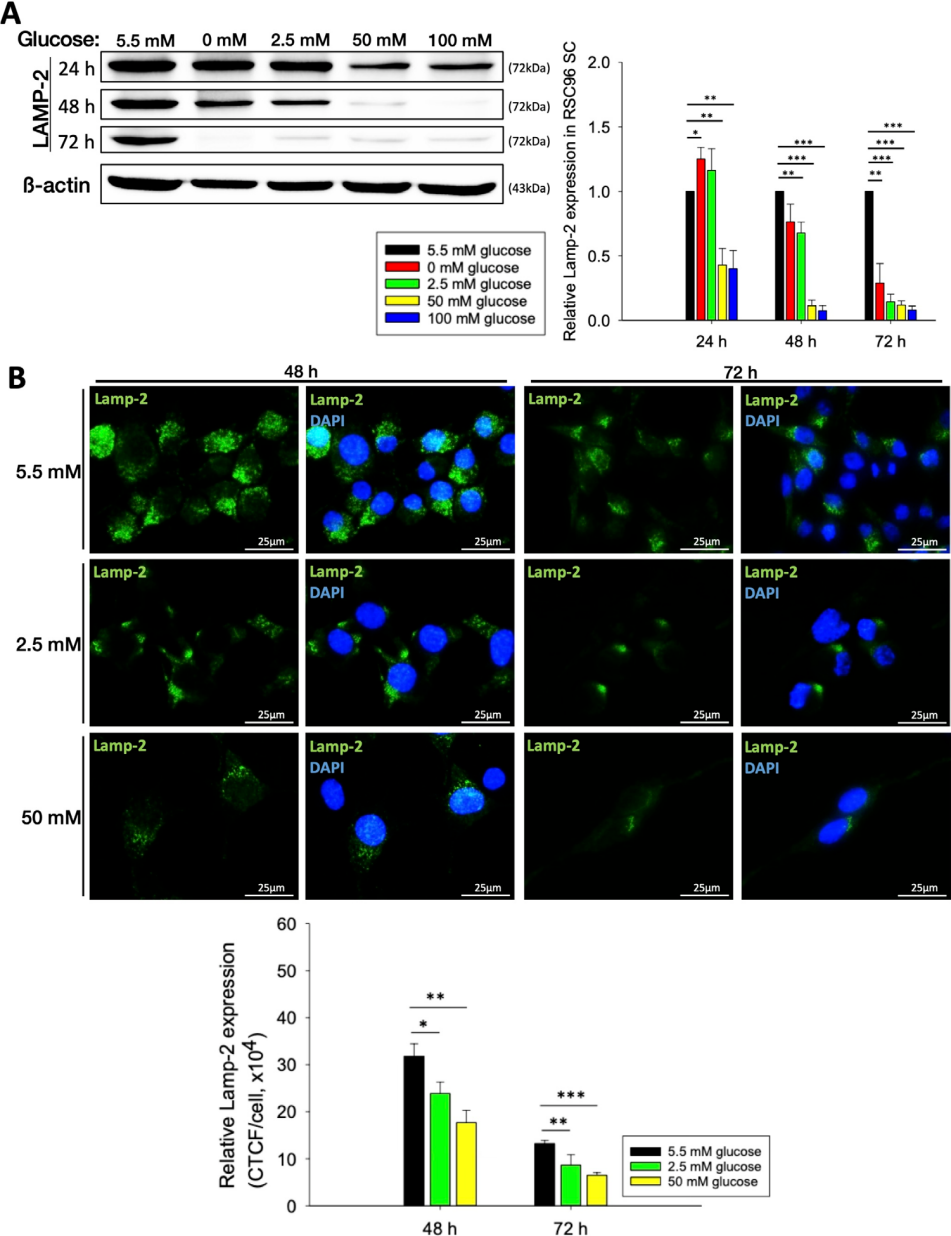
Reduced levels of Lamp‐2 protein expressions in Schwann cells under different glucose concentrations. (A) WB analyses, and (B) immunofluorescence staining of intracellular Lamp‐2 expressions (4× independent assays per group). Bars represent means ± SEs. Statistical analysis between the 5.5 mM glucose control group and each respective glucose treatment group per time point was performed via one‐way ANOVA, with *p < 0.05, **p < 0.01, ***p < 0.001. Scale bar: 25 μm.

To assess lysosomal fusion capacity, the expression of Lamp‐2 (lysosome‐associated membrane protein 2), a crucial transmembrane glycoprotein that facilitates lysosomal fusion events by providing structural support and membrane stability, and serving as a docking platform for fusion machinery components, was evaluated [ 11 ]. WB revealed significant and sustained reductions in Lamp‐2 levels under HG conditions at all time points (Figure 7A), with corresponding decreases in cytoplasmic Lamp‐2 by immunofluorescence (Figure 7B). In contrast, Lamp‐2 expression initially increased under glucose‐deprived and LG conditions at 24 h but then decreased at 48 and 72 h (Figure 7A), suggesting that late‐stage autophagy impairment occurred. Collectively, these results indicate that glucose deprivation and LG initially increase autophagy in SCs, which is impaired at 72 h because of reduced Lamp‐2 expression. In contrast, HG suppressed autophagic activity early, which coincided with sustained Lamp‐2 reduction and defective autophagosome–lysosome fusion.

## 4 Discussion

**FIGURE 8:**
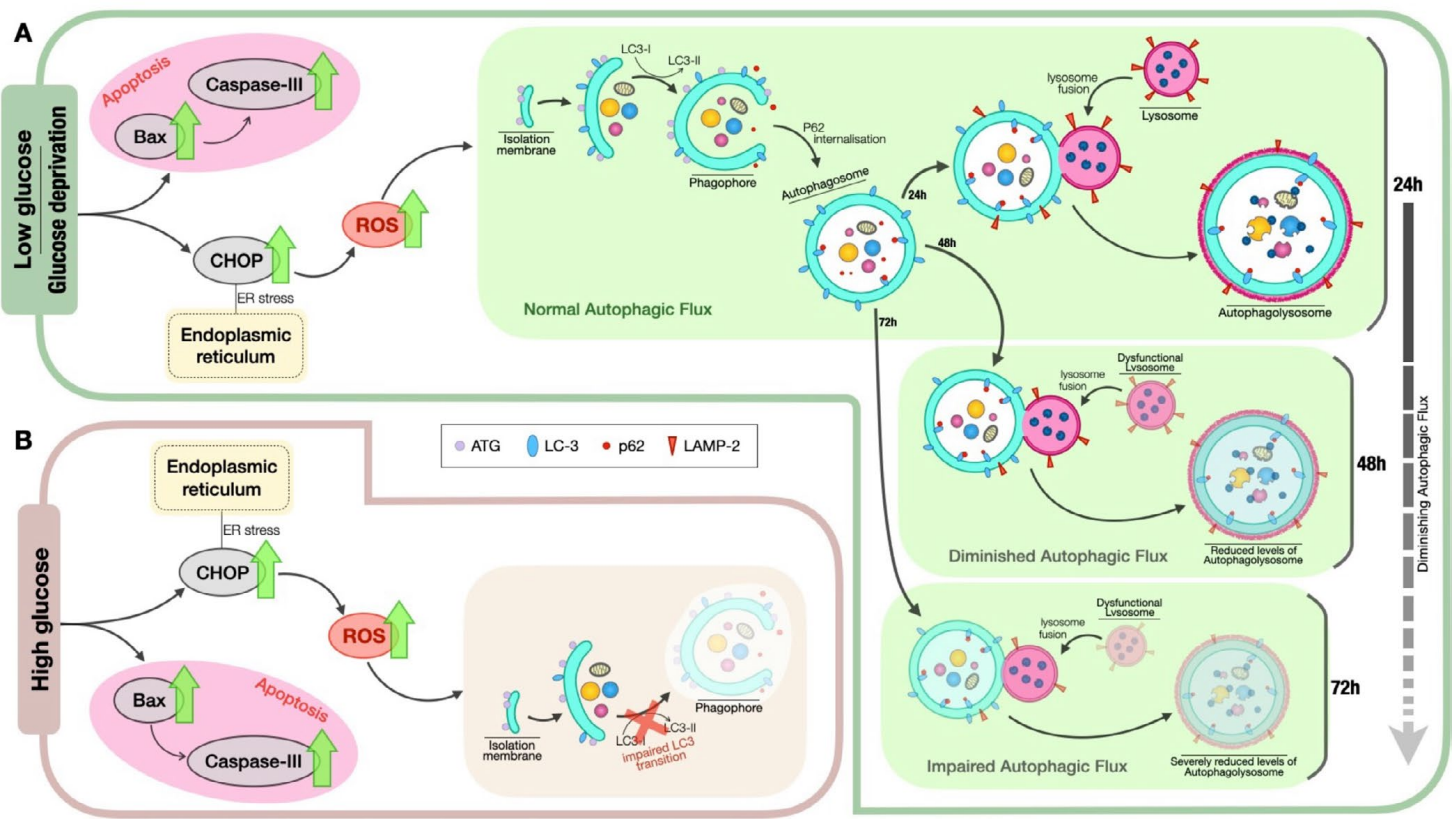
Schematic summary of differential patterns of autophagy impairment in Schwann cells under varying glucose conditions. The process of apoptosis, as well as increased levels of ROS was observed in rat Schwann cells cultured in both the (A) glucose‐limiting or glucose‐deprived conditions and (B) high glucose (HG) conditions. Both extremes of glucose imbalance triggered CHOP upregulation in RSC96 Schwann cells, leading to elevated ROS and attempted activation of autophagy. (A) Under glucose deprivation or low glucose (LG), autophagic flux was initially maintained but impaired by 72 h due to lysosomal dysfunction. (B) In contrast, HG conditions suppressed autophagy from the outset. These findings suggest that despite the initiation of stress responses, autophagy is ultimately dysfunctional under both low and HG conditions in RSC96 cells.

In addition to being responsible for forming myelin sheaths and supporting axonal function, SCs are also highly sensitive to glucose fluctuations. In this study, while glucose deprivation, LG, and HG conditions all induced oxidative stress and apoptosis in SCs, cell viability was significantly lower under glucose deprivation and LG conditions than under HG conditions, indicating a more severe cytotoxic effect in nutrient‐deficient environments. Importantly, the results of the present study demonstrated that the mechanisms underlying autophagy impairment differ greatly between low/deprived glucose and HG conditions. Under constant glucose deprivation and LG conditions, SCs initially responded to metabolic stress by upregulating autophagy as a survival mechanism. However, the subsequent downregulation of Lamp‐2 expression led to impairment of autophagosome–lysosome fusion, disrupting the completion of autophagic flux at later time points. In contrast, constant HG resulted in early autophagic dysfunction at the 24 h checkpoint with characteristic suppression of autophagy initiation and reduced Lamp‐2 expression, suggesting a direct inhibitory effect on the autophagic pathway. These findings highlight the dual vulnerabilities of SCs in either glucose‐deficient or glucose‐excessive environments, with each condition disrupting cellular homeostasis through distinct, yet detrimental, pathways (summarized in Figure 8).

Glucose is vital for cellular energy metabolism and homeostasis. In vitro hyperglycemia models typically use glucose concentrations between 30 and 150 mM (equivalent to 540–2700 mg/dL) [16], with 50 mM most frequently applied. Accordingly, in this study, 5.5 mM was used as the normal control condition, with 0 mM for glucose deprivation, 2.5 mM for LG, and 50 or 100 mM for HG conditions. The 100 mM high glucose group serves not only as the experimental extreme, but also to simulate the severe hyperglycaemic condition of patients suffering from diabetic ketoacidosis or diabetic hyperosmolar hyperglycemic syndrome [17]. DPN, a frequent complication of chronic hyperglycemia, is driven by oxidative stress linked to advanced glycation end‐products and impaired antioxidant defense [18].

Mitochondria and the ER are major sources of ROS under glucose stress [19]. In SCs, HG increases ROS levels, activates the NLRP3 inflammasome, and induces apoptosis [17]. Nox4, an NADPH oxidase, also promotes ROS production and apoptosis, with ROS detectable as early as 2 h in dorsal root ganglion neurons, emphasizing the rapid onset of oxidative stress and the need for early antioxidant intervention [20]. On the other hand, hypoglycemia, a common side effect of insulin or oral antidiabetic therapy, is increasingly linked to complications such as cardiovascular disease, nephropathy, neuropathy, and retinopathy [ 21 ]. Severe hypoglycemia can lead to structural and functional impairments in the central and peripheral nervous systems [22], and patients with a history of hypoglycemic episodes are at greater risk for dementia and irreversible brain damage [23].

Recurrent glucose fluctuations further induce oxidative stress and apoptosis in SCs, as evidenced by the upregulation of CHOP (an ER stress marker) expression and increased ROS levels [24]. Consistent with our present study, other reports have shown the upregulation of CHOP, ROS production, and apoptosis under strict glucose imbalance [10]. In SC studies, high glucose increased the expression of GRP78 and phosphorylated eIF2α and CHOP expression, whereas inhibition of ER stress effectively lowered the expression of CHOP and rescued cell viability [24]. However, the precise cellular mechanisms underlying the greater reduction in cell viability under glucose deprivation or low‐glucose conditions than under high‐glucose conditions remain unclear. Cui et al. reported that glucose deficiency depletes protons, leading to lysosomal deacidification and cell death [ 25 ], consistent with our observed reduction in Lamp‐2 expressions in RSC96 SCs. These findings suggest that prolonged energy depletion and disrupted glycolysis–lysosome–mitochondria interactions increase SC vulnerability under low‐glucose conditions.

Autophagy is crucial for cell survival and energy homeostasis, especially under stress, and its dysfunction is linked to diabetes and related diseases [11]. In this study, early autophagy impairment was observed in SCs under HG conditions, as evidenced by reduced LC3‐II/I ratios and LC3‐II‐positive puncta, whereas glucose deprivation or low glucose conditions caused a more gradual disruption. As autophagy is dynamic, assessing autophagic flux from initiation to lysosomal degradation is essential for distinguishing activation from blockage [26]. HG persistently blocked autophagic flux from 24 to 72 h, whereas the flux was initially activated under deprivation but impaired by 72 h, likely due to reduced Lamp‐2 expression and disrupted autophagosome–lysosome fusion. These findings highlight time‐dependent autophagy regulation under glucose stress and the need for thorough assessment. Although autophagy is well‐characterized in the CNS, its role in peripheral neuropathy remains unclear. Reduced expressions of LC3 and P62 in SCs under HG conditions aligned with observations reported in other cell types and in the sciatic nerves of DM murine models, possibly via JAK‐STAT3 signaling [27]. HG also inhibits autophagy and promotes apoptosis by suppressing the PI3K/Akt pathway and upregulating DNMT1, DNMT3a, and TXNIP expression [28]. However, there is opposing evidence of HG‐induced autophagy activation [29]. underscoring the need for further study.

In mammalian cells, AMP‐activated protein kinase (AMPK) regulates autophagy during glucose starvation by responding to reduced ATP levels, with mitochondrial ROS enhancing this process via AMPK modulation [30]. Energy stress and intermittent fasting also promote autophagy and support cell survival [31]. However, prolonged stress may trigger excessive autophagy, leading to type II autophagic cell death [32]. The dual role of autophagy remains debated, with evidence for both neuroprotection and neurotoxicity [33]. Our findings indicate that autophagy exceeding lysosomal capacity induces autophagic stress and cytotoxicity, as shown by early activation under glucose deprivation, followed by lysosomal dysfunction, reduced flux, and decreased cell viability. Other studies have indicated that AMPK–mTOR–ULK1‐driven autophagy initially acts as a pro‐survival buffer for the stressed cells under LG conditions. However, under sustained stress, JNK‐Bcl‐2/Beclin‐1 remodeling and proteolytic cleavage of ATG5/Beclin‐1 pivot the system toward apoptosis [34, 35]. Under HG conditions, ER stress, mitochondrial ROS, and mTOR/lysosomal constraints lead to an autophagy–apoptosis crossover characterized by defective flux and apoptosis [36].

Mitochondria are key ATP producers vital for neuronal function and survival, and are among the first targets affected by hypoglycemia, leading to ATP depletion and disrupted electrolyte balance [37]. Cardoso et al. further reported that insulin‐induced recurrent hypoglycemia worsened mitochondrial dysfunction and oxidative stress in the DM brain [38]. In this study, the reduced viability and impaired autophagy in SCs under glucose‐limited or glucose‐deprived conditions suggest that mitochondrial dysfunction is likely involved. However, the specific forms and extent of this dysfunction remain unclear. To clarify the effect of glucose imbalances on SCs' bioenergetics, future studies should investigate cell mitophagy, membrane potential, and ATP production under different glucose conditions.

In conclusion, persistent exposure of RSC96 SCs to high or low glucose levels led to increased cell death, which was driven primarily by elevated ROS levels and impaired autophagic flux through distinct mechanisms. HG suppressed autophagy early, causing sustained dysfunction, whereas glucose‐limited or glucose‐deprived conditions initially activated autophagy, followed by flux impairment likely due to Lamp‐2–related autophagosome–lysosome fusion failure. This is the first report to reveal differential patterns of autophagic disruption in SCs under opposing glucose stresses, offering new insight into glucose imbalance responses and potential therapeutic targets for DPN through autophagy modulation.

## Funding

This work was supported by the National Science and Technology Council, R.O.C, grant numbers MOST110‐2314‐B‐037‐091‐MY2 and MOST111‐2314‐B‐037‐098, as well as support from Kaohsiung Medical University Hospital research, Department of Medical Research grant number KMUH111‐1R79.

## Conflicts of Interest

The authors declare no conflicts of interest. All authors have read the journal's authorship agreement and policy on the disclosure or potential conflicts of interest. The authors also declared a familial relationship between Dr Yuan‐Chen Cheng and Dr Kuang‐I Cheng. Dr Yuan‐Chen Cheng has been under the tutelage of Dr Ling‐Li Chang, as well as being a longstanding member of the research group. This relationship did not influence the levels of members’ contributions, experimental design, data analysis, interpretation of results, or the objectivity of this study. The other authors declare that there are no relationships or activities that might bias, or be perceived to bias their work.

## Supporting information


**Figure S4:** Western blots results summary of p‐AKT in glucose (0 ~ 100 mM) treated RSC96 cells. Statistical analysis compared between designated groups performed via one‐way ANOVA, with *p < 0.05, **p < 0.01, ***p < 0.001.

## Data Availability

The data that support the findings of this study are available on request from the corresponding author. The data are not publicly available due to privacy or ethical restrictions.
