# Supplementary material for: Lysosome Evanescence Mediates Autophagic Flux Impairment in Glucose Imbalanced Environments
Source: Kaohsiung J Med Sci. 2025 Dec 13;42(7):e70145. doi: 10.1002/kjm2.70145 (PMC13344340; doi:10.1002/kjm2.70145)
Supplement: Supplementary file 1 — Figure S4: Western blots results summary of p‐AKT in glucose (0 ~ 100 mM) treated RSC96 cells. Statistical analysis compared between designated groups performed via one‐way ANOVA, with *p < 0.05, **p < 0.01, ***p < 0.001. [file KJM2-42-e70145-s001.docx]

**Supplementary data:**

**Supplementary Figure 4:** Western blots results summary of p-AKT in glucose (0~100 mM) treated RSC96 cells. Statistical analysis compared between designated groups performed via one-way ANOVA, with *p<0.05, **p<0.01, ***p<0.001.
